# Comparisons of N-glycans across invertebrate phyla

**DOI:** 10.1017/S0031182019000398

**Published:** 2019-05-03

**Authors:** Katharina Paschinger, Iain B. H. Wilson

**Affiliations:** Department für Chemie, Universität für Bodenkultur, 1190 Wien, Austria

**Keywords:** Glycomics, glycosyltransferase, N-glycan

## Abstract

Many invertebrates are either parasites themselves or vectors involved in parasite transmission; thereby, the interactions of parasites with final or intermediate hosts are often mediated by glycans. Therefore, it is of interest to compare the glycan structures or motifs present across invertebrate species. While a typical vertebrate modification such as sialic acid is rare in lower animals, antennal and core modifications of N-glycans are highly varied and range from core fucose, galactosylated fucose, fucosylated galactose, methyl groups, glucuronic acid and sulphate through to addition of zwitterionic moieties (phosphorylcholine, phosphoethanolamine and aminoethylphosphonate). Only in some cases are the enzymatic bases and the biological function of these modifications known. We are indeed still in the phase of discovering invertebrate glycomes primarily using mass spectrometry, but molecular biology and microarraying techniques are complementary to the determination of novel glycan structures and their functions.

## Introduction

The co- or post-translational addition of glycans to proteins takes various forms in all kingdoms of life (Varki, 2011); amongst the most common is N-glycosylation, by which asparagine residues are modified. In eukaryotes, most commonly a Glc_3_Man_9_GlcNAc_2_ precursor is transferred from dolichol to proteins in the endoplasmic reticulum (Aebi, 2013); however, some protists utilise shorter precursors or even do not N-glycosylate at all (Samuelson *et al*., 2005). The fates of protein-linked N-glycans are varied and depend on the types of glycosidases and glycosyltransferases expressed in the Golgi apparatus. It is this variability that makes glycan analysis a challenge, as so many possibilities occur by which N-glycans are trimmed and then built up again.

Other than the first steps in the endoplasmic reticulum, the final size and form of N-glycans differ between protists, fungi, plants and animals (whether invertebrate or vertebrate), although some modifications are found in more than one of these groups of organisms. Unlike plants whose N-glycomes are similar from mosses through to *Arabidopsis*, there is high variability between non-vertebrate eukaryotes (Schiller *et al*., 2012). Here, we will concentrate on primarily structural aspects of invertebrate N-glycans, not only due to the parasitological relevance (as many invertebrates are either hosts, vectors or themselves parasites), but also because only recently have mass spectrometric analyses revealed a previously unrealised range of modifications, some of which are shared with O- and lipid-linked glycans. A few years ago, one would probably have read that invertebrates only produce oligomannosidic (Man_5–9_GlcNAc_2_) and paucimannosidic (Man_1–4_GlcNAc_2_Fuc_0–2_) N-glycans (Williams *et al*., 1991); this may be due to insensitive methods and low expectations, but it is now known that even complete glycomes of some mammalian cell types are dominated by oligomannosidic forms present within the secretory pathway (Hamouda *et al*., 2014).

## Oligomannosidic N-glycans

**Fig. 1.**
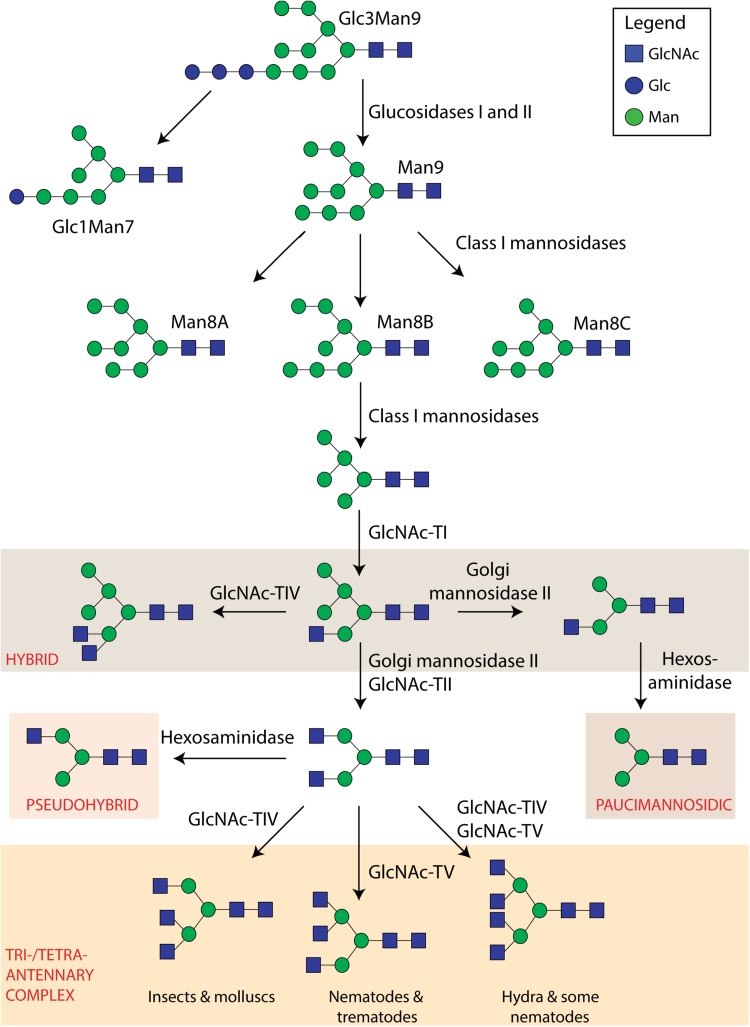
Simplified biosynthetic scheme for N-linked glycans in animals. Starting with the Glc_3_Man_9_GlcNAc_2_ precursor, various glycosidases result in different isomers of oligomannosidic glycans with the maximal degree of processing by class I mannosidases yielding Man_5_GlcNAc_2_. This is the substrate for *N*-acetylglucosaminyltransferase I (GlcNAc-TI) which generates a ‘hybrid’ structure which can be further modified by the action of Golgi mannosidase II, GlcNAc-TII and Golgi hexosaminidase. The maximum number of antennae (three or four) depends on the presence of GlcNAc-TIV and GlcNAc-TV; example hybrid, pseudohybrid, paucimannosidic and tri-/tetra-antennary glycans are shown as known from various model, host, vector or parasitic invertebrates. For simplicity, fucosylation and other modifications are not included. Glycans are depicted according to the Standard Nomenclature for Glycans (see also box).

Even within the glycans containing primarily mannose residues (hence oligomannosidic or high mannose), there is variation arising from the different orders of processing by so-called class I *α*1,2-mannosidases, also in parasitic metazoa. Most eukaryotes have multiple forms of these *α*1,2-mannosidases (Wilson, 2012), which also include enzymes known as EDEMs (ER degradation-enhancing *α*-mannosidases) acting as part of the quality control pathway in the endoplasmic reticulum. The result is that there are multiple isomers of oligomannosidic structures (e.g. three isomers of glycans with the composition Man_8_GlcNAc_2_; Fig. 1) just depending on which mannosidase acts first on particular terminal mannose residues; the final product is the ‘Golgi’ isomer of Man_5_GlcNAc_2_. These structures can be differentiated by, e.g. RP-HPLC in combination with MS/MS and thus it is only appropriate to annotate specific isomers based on such information; for instance, a Hex_8_HexNAc_2_ structure could also be Glc_1_Man_7_GlcNAc_2_ and not necessarily one of three typical forms of Man_8_GlcNAc_2_. Oligomannosidic glycans may also be the ‘final’ processed forms in the cases where protein folding prevents a specific glycosylation site from being accessible to enzymes in the Golgi apparatus (Thaysen-Andersen and Packer, 2012). On the basis of the universality of oligomannosidic glycans in metazoa, it is not surprising that these glycans have been observed in a wide range of invertebrates including trematodes, nematodes, molluscs and insects.

## Hybrid and pseudohybrid N-glycans

The classical ‘hybrid’ structure is a ‘Golgi-type’ Man_5_GlcNAc_2_ modified on the ‘lower’ *α*1,3-mannose by *β*1,2-specific *N*-acetylglucosaminyltransferase I (GlcNAc-TI; encoded by the mammalian MGAT1 gene and its homologues in multicellular eukaryotes, including parasitic invertebrates) to yield Man_5_GlcNAc_3_ (Fig. 1) which may be the substrate for further modification. This is a key intermediate in N-glycan biosynthesis (Fig. 1) in terms of the routes to processed structures as well as in biological terms (Schachter, 2010), as ablation of this gene results in large glycomic shifts as well as a range of phenotypes (Shi *et al*., 2006; Sarkar *et al*., 2010; Yan *et al*., 2018*b*), most dramatically, the embryonic lethal phenotype in mammals (Ioffe and Stanley, 1994; Metzler *et al*., 1994).

Once GlcNAc-TI has acted, Golgi mannosidase II will remove one or two of the ‘upper’ mannose residues and also represents a potential biosynthetic bottleneck (Paschinger *et al*., 2006). If there is no transfer by GlcNAc-TII thereafter to the *α*1,6-mannose, then the glycan remains in a hybrid state (i.e. sharing aspects of oligomannosidic and complex structures); also even if GlcNAc-TIV (*β*1,4-specific) modifies the *α*1,3-mannose, then the glycan is still classified as being hybrid (Kornfeld and Kornfeld, 1985). The lower arm *β*1,2- and *β*1,4-GlcNAc residues on hybrid glycans can be modified in different ways; elongation by *β*1,4-*N-*acetylgalactosamine and *β*1,3- or *β*1,4-galactose are known in insects, molluscs, nematodes and trematodes, whether these be host or parasitic organisms (Nyame *et al*., 1989; Kurz *et al*., 2013, 2015; Martini *et al*., 2019; Smit *et al*., 2015). If however, GlcNAc-TII acts and then the ‘lower’ arm *β*1,2-GlcNAc, transferred by GlcNAc-TI, is removed by a Golgi hexosaminidase such as *fdl* (fused lobes) in insects or HEX-2 in nematodes (Gutternigg *et al*., 2007*b*; Geisler and Jarvis, 2012), then the resulting glycans can be referred to as ‘pseudohybrid’. Such structures are also found in protist parasites lacking GlcNAc-TI, but having GlcNAc-TII-like enzyme activities (Paschinger *et al*., 2012*b*; Damerow *et al*., 2014). The core of hybrid glycans in animals can also be modified, most commonly by *α*1,6-fucose.

## Paucimannosidic N-glycans

The term ‘paucimannosidic’ glycans was introduced to cover those glycans which have been processed serially by GlcNAc-TI, Golgi mannosidase II and a Golgi hexosaminidase to result in Man_3–4_GlcNAc_2_ (Gutternigg *et al*., 2007*b*). Such structures are well known in invertebrates and plants, but also occur due to the action of acidic glycosidases on glycoproteins in the secretory granules in some mammalian cells (Loke *et al*., 2017). A significant portion of paucimannosidic glycans are core fucosylated and carry the ‘mammalian-like’ *α*1,6-fucose and the ‘plant-like’ *α*1,3-fucose either alone or in combination on the reducing-terminal (proximal) GlcNAc of the core region of the N-glycan as found first on bee venom glycoproteins (Kubelka *et al*., 1993). In nematodes, the second (distal) GlcNAc can also be modified (Haslam *et al*., 1996; Hanneman *et al*., 2006).

**Fig. 2.**
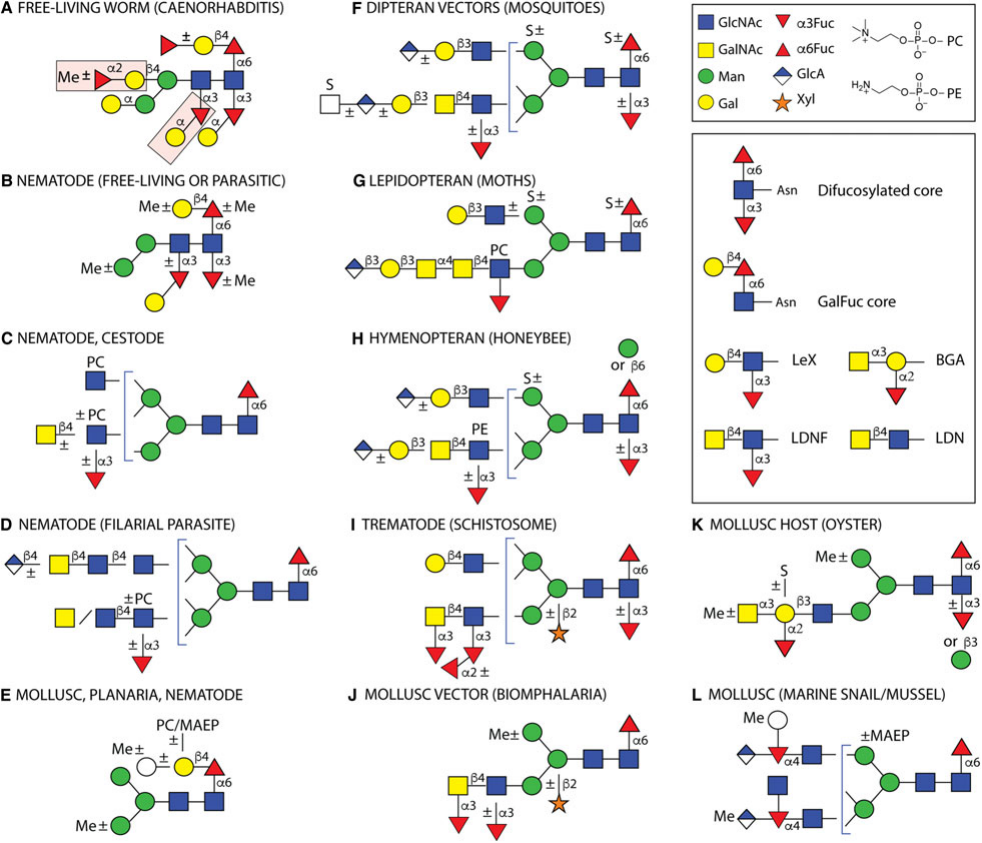
Example N-glycans from invertebrates. Structures are depicted from either parasitic or free-living organisms, whereby some of the latter are hosts or vectors for parasites. Some types of structures are species- or class-specific, but others are found in more than one phylum. Only a non-exhaustive selection of core and antennal epitopes is shown in the inset: core difucosylation, core ‘GalFuc’, Lewis X (LeX), fucosylated and non-fucosylated LacdiNAc (LDN) and blood group A (BGA). (A) The bisecting and distal core modifications found in the free-living *C. elegans* are indicated by pink boxes; (B) free-living *C. elegans*, the necromenic *P. pacificus* and the parasites *H. contortus*, *H. polygyrus* and *O. dentatum* express di- and/or tri-fucosylated cores with species-specific galactosylation and methylation; (C) varying antennal modifications are found in all nematodes as well as the cestodes *E. granulosus* and *T. crassiceps*, (D) while filarial species have up to four long antennae including *D. immitis,* which has in addition glucuronylated structures; (E) galactosylated core fucose (GalFuc) is found in many invertebrates, sometimes in substituted form; (F, G and H) selected complex glycans from larvae of different insect phyla; (I) selected *S. mansoni* N-glycan modifications which are partly stage-specific; (J, K and L) selected gastropod and bivalve glycans, including those of *Crassostrea virginica*, *B. glabrata*, *Volvarina rubella* and *Mytilus edulis*. Note that some modifications, such as core *β*-mannosylation, are at low abundance in the relevant glycomes. Glycans are depicted according to the Standard Nomenclature for Glycans; undefined hexoses/*N-*acetylhexosamines are shown as white circles/squares. Me, methyl; MAEP, *N-*methyl-aminoethylphosphonate; PC, phosphorylcholine; PE, phosphoethanolamine (2-aminoethylphosphate); S, sulphate. Broken lines,±or brackets indicate structure-, species- or stage-dependent variations in these elements.

Both proximal core *α*1,3-fucose and substitution of the *β*-mannose by *β*1,2-xylose (see Fig. 2 for example structures) are immunogenic in mammals and antibodies raised against plant and invertebrate glycoproteins often recognise these epitopes, the best known example of which is anti-horseradish peroxidase (anti-HRP); both structural elements are epitopes for IgE or IgG in parasite-infected animals or children as well as in individuals allergic to plant pollen, food or insect venom, although the clinical relevance is controversial (van Die *et al*., 1999; Altmann, 2007; Paschinger *et al*., 2009; Brzezicka *et al*., 2015; Amoah *et al*., 2018). While core *α*1,3-fucose is widespread in invertebrates, xylosylation of N-glycans is known from gastropods and, in a stage-specific manner, *Schistosoma* spp. (Khoo *et al*., 1997; Gutternigg *et al*., 2007*a*; Lehr *et al*., 2010; Smit *et al*., 2015). This is interesting as some gastropods (specifically snails such as *Biomphalaria glabrata*) are intermediate hosts for schistosomes. The activities of core-modifying fucosyl- and xylosyltransferases have been detected in extracts of various species, but only for core *α*1,3/*α*1,6-difucosylation have relevant genes been identified and recombinant forms of the enzymes characterised (Fabini *et al*., 2001; Paschinger *et al*., 2005; Rendić *et al*., 2007; Kurz *et al*., 2016).

## Modified N-glycan cores

In addition to fucosylation and xylosylation, some invertebrates attach further monosaccharide units to the basic paucimannosidic core. Recently joining the list of core modifications alongside galactosylation of core *α*1,6-fucose (‘GalFuc’), first detected in squid and then in keyhole limpet, planaria and nematodes (Takahashi *et al*., 2004; Wuhrer *et al*., 2004; Titz *et al*., 2009; Paschinger *et al*., 2011; Subramanian *et al*., 2018), are *α*-galactosylation of the proximal and distal core *α*1,3-fucose residues (Yan *et al*., 2018*a*), elongation of the GalFuc unit by galactose (Wuhrer *et al*., 2004; Subramanian *et al*., 2018), fucose (Yan *et al*., 2015*b*), phosphorylcholine or methylaminoethylphosphonate (Eckmair *et al*., 2016), *β*-mannosylation of the proximal GlcNAc (Eckmair *et al*., 2016; Hykollari *et al*., 2018) and the galactosylation of the core *β*-mannose to form a bisected structure, which can also be modified by methylated or nonmethylated fucose (Yan *et al*., 2015*a*) (Fig. 2). While the latter bisecting modifications have only been found in the non-parasitic nematode *Caenorhabditis elegans*, the zwitterionic modifications of the GalFuc have been detected uniquely in a marine gastropod; however, galactosylation of the proximal *α*1,6-fucose and distal *α*1,3-fucose residues has also been found in the parasitic nematodes *Oesophagostomum dentatum* and *Haemonchus contortus* (Paschinger and Wilson, 2015; Sutov, 2016; Jiménez-Castells *et al*., 2017).

Some of the reason for the apparent restriction in what is found might be methodological. For instance, the presence of *α*-galactose on the proximal *α*1,3-fucose was only detected in *C. elegans* when using hydrazine or the newly-developed PNGase Ar enzyme to release the N-glycans, whereby the maximal degree of core fucosylation in this worm (five fucoses) was only found after hydrazinolysis (Yan *et al*., 2018*a*). Only in the case of *O. dentatum* can we say that these modifications are absent, since hydrazinolysis was also performed with samples from this organism and MS/MS did not reveal any glycan with the relevant fragmentation pattern (Jiménez-Castells *et al*., 2017). On the other hand, *H. contortus* glycans were only ever analysed after ‘classical’ PNGase F and A digestion and so it can only be speculated as to whether it shares more complex cores with *C. elegans.*

The enzymatic basis for only some of these modifications is known. Three core-modifying *α*-fucosyltransferases (FUT-1, FUT-6 and FUT-8) are known from *C. elegans* as is the *α*1,6-fucose-modifying GALT-1 galactosyltransferase from the same organism (Paschinger *et al*., 2004, 2005; Titz *et al*., 2009; Yan *et al*., 2013). The *in vitro* activity data is complemented by glycomic studies on mutants showing the absence of the relevant epitopes (Butschi *et al*., 2010; Yan *et al*., 2015*b*). Some of these glyco-mutants have altered susceptibility to nematoxic fungal lectins (Butschi *et al*., 2010; Schubert *et al*., 2012), which are also toxic to *H. contortus* (Heim *et al*., 2015). For all the other modifications around the core, e.g. the addition of various *α*-galactose residues or of bisecting *β*-galactose in *C. elegans* we have no clues as to which enzymes may be responsible. The same lack of knowledge applies to *β*-mannosylation of the proximal core GlcNAc in molluscs and insects.

## Complex N-glycans

The definition ‘complex N-glycan’ is based on the knowledge of mammalian glycosylation and refers to glycans with at least one GlcNAc modifying both *α*-mannose residues of the trimannosyl core. Thus, both GlcNAc-TI and -TII (MGAT1 and MGAT2) have acted and these can be supplemented by GlcNAc-TIV, GlcNAc-TV and in some species ‘GlcNAc-TVI’ (Schachter, 1986). The other common *N*-acetylglucosaminyltransferase, GlcNAc-TIII, is a bisecting enzyme found in vertebrates. The result of the action of these various enzymes (see Fig. 1) is the various bi-, tri- and tetra-antennary glycans (even penta-antennary in birds and fish), which are well known from the serum glycomes of mammals.

It may come as a surprise that even relatively primitive animals have tri- or tetra-antennary N-glycans as found in *Hydra,* molluscs, insects and nematodes (Kang *et al*., 1993; Morelle *et al*., 2000; Kurz *et al*., 2013, 2015; Sahadevan *et al*., 2014; Eckmair *et al*., 2016). The exact nature of the tri-antennary glycans varies, as GlcNAc-TIV products occur in molluscs and insects (Kurz *et al*., 2013, 2015), but GlcNAc-TV acts in glycan biosynthesis in a number of nematodes such as *C. elegans* and *Pristionchus pacificus* (Yan *et al*., 2015*c*). Other nematodes, though, do have both GlcNAc-TIV and -TV homologues and so can have up to four branches on their N-glycans as found in filarial species or in *Trichinella* (Haslam *et al*., 1999; Kang *et al*., 1993; Morelle *et al*., 2000; Martini *et al*., 2019).

Amongst trematodes, triantennary glycans have been long established to exist in *S. mansoni* males (Nyame *et al*., 1989); it has also been suggested that up to four branches may also be on N-glycans of *S. mansoni* eggs or in *Opisthorchis viverrini* (Talabnin *et al*., 2013; Smit *et al*., 2015); however, as in another trematode *Fasciola hepatica* (McVeigh *et al*., 2018), BLAST searching of the available genomes only shows an obvious GlcNAc-TV homologue and none of GlcNAc-TIV (unpublished data). However, only for *C. elegans* GlcNAc-TI and GlcNAc-TII is there *in vitro* evidence from recombinant enzymes to verify the predicted activities (Chen *et al*., 2002), while *C. elegans* GlcNAc-TV has been shown to complement a relevant Chinese hamster ovary mutant cell line in terms of lectin sensitivity (Warren *et al*., 2002); the activity of an invertebrate GlcNAc-TIV has still to be proven.

Subsequent to the initial transfer of up to four non-reducing terminal GlcNAc residues, further elongation events can occur and these are extremely variable and, in non-vertebrates, include substitutions with *β*1,3-galactose, *β*1,4-*N-*acetylgalactosamine, *α*1,4-*N-*acetylgalactosamine or fucose as well as anionic, zwitterionic or methyl groups (Fig. 2). The typical mammalian form of galactosylation (*β*1,4) is not so widespread in lower animals in general, but it can, e.g. be found in *Schistosoma* spp. (Khoo *et al*., 1997; Smit *et al*., 2015); it can only be distinguished from *β*1,3-galactosylation by use of specific galactosidases and, if amounts allow, by NMR spectroscopy or GC-MS methods. Antennal GlcNAc residues modified with *β*1,3Gal or even *β*1,3Gal*β*1,4GalNAc are found on N-glycans from, e.g. mosquitoes acting as intermediate hosts for parasites and viruses (Kurz *et al*., 2015) and *β*1,3-Gal is also found in the oyster *Crassostrea virginica*, which is a host for the *Perkinsus marinus* protist parasite (Kurz *et al*., 2013). On the other hand, GalNAc*β*1,4GlcNAc (LacdiNAc) is a known motif from various insects and nematodes (see also the section on fucosylated antennae below) and a stage-specific bias in its expression is known from trematode parasites (Talabnin *et al*., 2013; Smit *et al*., 2015). Longer chito-based (GlcNAc*β*1,4GlcNAc) antennae are a feature of filarial nematodes as well as of *H. contortus* and *O. dentatum* (Haslam *et al*., 1999; Sutov, 2016; Jiménez-Castells *et al*., 2017; Martini *et al*., 2019).

For some of these terminal modifications, the relevant enzymes have been identified and characterised in recombinant form, such as *β*1,4-*N-*acetylgalactosaminyltransferases from *C. elegans* and *Trichoplusia ni*, a *β*1,3-galactosyltransferase from the honeybee and an *α*1,4-*N-*acetylgalactosaminyltransferase from *Drosophila* (Kawar *et al*., 2002; Mucha *et al*., 2004; Vadaie and Jarvis, 2004; Ichimiya *et al*., 2015). However, although some relevant enzyme activities have been detected in crude extracts, the identities of relevant genes in parasites are yet to be established.

## Antennally fucosylated N-glycans

Fucose as a deoxyhexose rather than a standard hexose may well, due to its chemical properties, be pre-destined to act as a recognition element. Indeed, fucose is the basis for mammalian histo-blood group antigens such as ABO and Lewis motifs (Fig. 2). Fucosylated LacNAc (Le^x^) and LacdiNAc (LDNF) epitopes are well known from *S. mansoni* (Khoo *et al*., 1997; Wuhrer *et al*., 2006; Smit *et al*., 2015) and may contribute to the lectin-dependent immunomodulatory activity of secreted schistosome proteins (Wilbers *et al*., 2017). Also, some nematodes (e.g. *Dictyocaulus viviparus, Trichuris suis* or *H. contortus*) and insects (e.g. the honeybee) express these epitopes (Kubelka *et al*., 1993; Haslam *et al*., 2000; Paschinger and Wilson, 2015; Wilson and Paschinger, 2016), while fucosylated chito-oligomers are a feature of the antennae of some N-glycans from *Dirofilaria immitis* (Martini *et al*., 2019) and Fuc*α*1,3GlcNAc as a terminal motif is also known from the cestode *Taenia crassiceps* (Lee *et al*., 2005). Less familiar may be the occurrence of blood group A on oyster glycans (Kurz *et al*., 2013), which are probable ligands for noroviruses in the marine environment, but which are also recognised by the oyster's own galectins (Feng *et al*., 2013). Interestingly, though, these galectins also mediate entry of *P. marinus* into oyster haemocytes, despite the apparent lack of blood group antigens on the parasite.

Generally, fucose on glycan antennae is unsubstituted, but branched fucose (i.e. disubstituted) is known in some molluscs (Zhou *et al*., 2013; Eckmair *et al*., 2016) and fucosylated fucose (Fuc*α*1,2Fuc*α*1,3) occurs in *S. mansoni* (Jang-Lee *et al*., 2007; Smit *et al*., 2015) (Fig. 2). Interestingly, the various fucosylated antennal modifications of *S. mansoni* are epitopes for various natural and monoclonal antibodies (van Remoortere *et al*., 2000; van Diepen *et al*., 2012) and may mediate interactions of parasitic proteins with cells of the host immune system (Meevissen *et al*., 2012). The schistosome genome encodes a number of fucosyltransferases, but only one has proven enzymatic activity in recombinant form, specifically as a Le^X^ synthase (Mickum *et al*., 2016*b*). Other defined invertebrate Lewis-type fucosyltransferases include the FucTC from the honeybee and a mosquito (Kurz *et al*., 2016; Rendić *et al*., 2007).

## Methylated N-glycans

Substitution of glycans by methyl groups is known in bacteria, plants and invertebrates. In the case of N-glycans from mollusc, planaria and free-living or parasitic nematodes, examples include methylation of mannose, fucose, galactose and *N-*acetylgalactosamine residues (van Kuik *et al*., 1986, 1987*b*; Gutternigg *et al*., 2007*a*; Paschinger *et al*., 2011; Kurz *et al*., 2013; Hewitson *et al*., 2016; Jiménez-Castells *et al*., 2017; Yan *et al*., 2018*a*) (Fig. 2). If analysing the glycans using standard permethylation conditions, such natural methyl groups are lost; thus, perdeuteromethylation has to be employed (Wohlschlager *et al*., 2014). For standard exoglycosidase sequencing, methylation normally prevents removal of a residue, but the methylated GalNAc on oyster glycans could be removed with chicken *α*-*N-*acetylgalactosaminidase (Kurz *et al*., 2013), while methylated *α*1,2- or *α*1,3-fucose residues on nematode glycans can be partially or fully released by hydrofluoric acid treatment (Yan *et al*., 2018*a*). The type of methylation can also vary within a species, as methylation of mannose was more common in male *O. dentatum* parasites as opposed to the methylfucose residues found in the female (Jiménez-Castells *et al*., 2017).

## Glucuronylated and sialylated N-glycans

By separating neutral from anionic glycans early in the analyses, we have been able to find glucuronic acid on the termini of N-glycans from a number of species, including mosquitoes, moths and the honeybee, as well as a marine snail (Kurz *et al*., 2013, 2015; Eckmair *et al*., 2016; Stanton *et al*., 2017; Hykollari *et al*., 2018) (Fig. 2). Like methylated hexose residues, the presence of glucuronic acid results in a mass increment of 176 Da, but GlcA-containing glycans can be detected by negative mode mass spectrometry (Hykollari *et al*., 2017). Using permethylation, others also detected glucuronic acid on N-glycans of *Drosophila* (Aoki and Tiemeyer, 2010), whereas we have also used glucuronidases to help prove its occurrence on oligosaccharide structures from other insects (Stanton *et al*., 2017; Hykollari *et al*., 2018). Except for *Dirofilaria immitis* (Martini *et al*., 2019), there are no reports to date of GlcA on N-glycans of nematodes or trematodes, but glycosaminoglycan chains and O-glycans from these species do contain this residue (Palaima *et al*., 2010; Vanbeselaere *et al*., 2018), including the circulating anodic antigen of *S. mansoni* (Bergwerff *et al*., 1994).

As glucuronic acid is a major component of glycosaminoglycans and these are known to play roles in host-pathogen interactions (Pinzon-Ortiz *et al*., 2001; Armistead *et al*., 2011), one can speculate that glucuronic acid on N-glycans may be another ligand involved in, e.g. *Plasmodium* transmission by mosquitoes. The role of glucuronylation of *Dirofilaria* N-glycans is also unclear. The actual transfer of glucuronic acid to N-glycans has not been proven for any invertebrate glucuronyltransferase, other than for two enzymes of broad specificity from *Drosophila* (Kim *et al*., 2003).

In terms of sialylation, for which there is no hint in most invertebrates, its occurrence in insects has been controversial. Other than mass spectrometric studies on N-glycans from *Drosophila* embryos (Aoki *et al*., 2007; Frappaolo *et al*., 2017), there is no firm proof to date for sialylation in any other insect; this is despite genome sequencing typically indicating the presence of one sialyltransferase homologue per insect species, some of which have proven *in vitro* activities (Koles *et al*., 2004; Kajiura *et al*., 2015). Higher up the evolutionary tree, however, there is good evidence for sialic acids on the O-glycans of Echinodermata (Miyata *et al*., 2006). Lectin binding data, although suggestive, is too ambiguous to be considered proof of the presence of sialic acid in unknown glycomes, as the ‘summarised’ specificities of many lectins are probably a simplification, but also contamination must be considered if detecting sialylation in glycans of a parasite derived from a mammalian host.

## Sulphated and phosphorylated N-glycans

Another surprisingly widespread anionic modification is sulphate, which results in signals in negative mode mass MS and a Δm/z of 80 mass units. Thereby, for many instruments, sulphate cannot be differentiated from phosphate; however, some very high resolution mass spectrometers can be used to distinguish these. Other proofs include the ionisation of phosphate in both positive and negative mode or the susceptibility of phosphate (and not of sulphate) to hydrofluoric acid or phosphatase treatments (Hykollari *et al*., 2017). By pre-separating neutral and anionic glycans prior to off-line LC-MS, we have detected sulphate in marine molluscs (including oyster) and in insects (including mosquitoes). On the other hand, standard permethylation procedures will result in loss of sulphated glycans, but modified solid phase extraction methods are compatible with subsequent detection of permethylated sulphated glycans as performed with mosquito or royal jelly N-glycans (Kurz *et al*., 2015; Hykollari *et al*., 2018).

Sulphation of invertebrate N-glycans may occur at different positions, e.g. of mannose or core fucose in arthropods or of galactose as in oyster (van Kuik *et al*., 1987*a*; Kurz *et al*., 2013, 2015) (Fig. 2), but we have yet to definitely prove sulphation in a parasite. Others have detected phosphorylation of mannose residues in *F. hepatica* (Ravida *et al*., 2016). The mannose-6-phosphorylation system known for trafficking of lysosomal enzymes in vertebrates is not proven in any invertebrate; strangely, though, a mannose phosphorylation mediated by a homologue of the relevant GlcNAc-1-phosphotransferase enzyme is found in an amoeba (Qian *et al*., 2010). There is no information regarding any N-glycan-modifying sulpho- or phosphotransferase from any invertebrate.

## Zwitterionic N-glycans

Phosphodiester and phosphonate modifications such as phosphorylcholine, phosphoethanolamine and aminoethylphosphonate may be familiar to many from bacterial lipopolysaccharides and glycosylphosphatidylinositol anchors or related molecules, but have been reported on a number of invertebrate N-, O- and lipid-linked glycans. While detection of these modifications is incompatible with permethylation procedures, they can all be released with hydrofluoric acid (HF) and so some earlier reports for their presence were based partly on detection of permethylated forms of ‘stripped’ glycans as well as of perdeuteroacetylated structures without HF treatment (Haslam *et al*., 1999; Morelle *et al*., 2000). However, when conducting more ‘native’ mass spectrometric analyses, phosphorylcholine (PC; Δm/z 165 mass units) ionises very well in positive mode and is a widespread modification of nematode N-glycans (Hanneman *et al*., 2006; Pöltl *et al*., 2007; Paschinger and Wilson, 2015; Hewitson *et al*., 2016; Wilson and Paschinger, 2016; Jiménez-Castells *et al*., 2017; Martini *et al*., 2019), but has also been found in a cestode (*Echinococcus granulosus*) and more recently on moth N-glycans (Paschinger *et al*., 2012*a*; Stanton *et al*., 2017) (Fig. 2).

Phosphoethanolamine (PE; Δm/z 123), aminoethylphosphonate (AEP; Δm/z 107) and methylaminophosphonate (MEAP; Δm/z 121) are detected in both positive and negative modes (Paschinger and Wilson, 2016). PE is found on N-glycans of royal jelly, AEP on those of a locust glycoprotein and MEAP on the antennae and core regions of N-glycans from a marine snail (Hård *et al*., 1993; Eckmair *et al*., 2016) (Fig. 2); other reports have shown PC, PE and MEAP on glycolipids or O-glycans of various invertebrates, including *Ascaris suum* (Hayashi and Matsubara, 1989; Sugita *et al*., 1992; Lochnit *et al*., 1998; Seppo *et al*., 2000; Maes *et al*., 2005; Urai *et al*., 2009).

PC and PE are ligands for pentraxins and so binding of *Echinococcus* Ag5 or of *Dirofilaria* glycans to C-reactive protein or of royal jelly N-glycans to serum amyloid P have been shown (Paschinger *et al*., 2012*a*; Hykollari *et al*., 2018; Martini *et al*., 2019). On the other hand, PC modifications of glycoconjugates are associated with immunomodulation; a well-known example of this being the ES-62 excretory-secretory protein from the filarial worm *Acanthocheilonema viteae* (Pineda *et al*., 2014). The biosynthesis of zwitterionic N-glycans remains unresolved, other than a requirement for the prior action of GlcNAc-TI in *C. elegans* (Houston *et al*., 2008), but comparisons with pathways in bacteria and fungi may help in the future to decipher the molecular basis for these reactions.

## N-glycan arrays

Glycans mediate function when they can be recognised and glycan arrays have become an established method for determining which proteins can bind them. However, other than *S. mansoni* (van Diepen *et al*., 2012; Mickum *et al*., 2016*a*), studies using natural structures are in their relative infancy for invertebrates, but pools or fractions of natural N-glycans from royal jelly, *Dirofilaria* and *C. elegans* have been tested recently in an immobilised format with pentraxins, selected antibodies or standard lectins (Hykollari *et al*., 2018; Jankowska *et al*., 2018; Martini *et al*., 2019). The bias in the literature towards schistosome arrays is probably due to a number of factors, such as availability of the various stages of the life-cycle and of monoclonal antibodies as well as three decades of relevant glycomic research. Thus, it has been possible to construct arrays of N-, O- and lipid-linked glycans derived from different stages of the schistosome life-cycle and screen them, e.g. with antibodies or antisera (van Diepen *et al*., 2012, 2015; Yang *et al*., 2017, 2018). Otherwise, some anti-helminth antibody responses have been tested against the primarily mammalian array of the Consortium for Functional Glycomics, remodelled glycans or conjugates with shorter saccharides to identify potential protective or diagnostic epitopes (van Stijn *et al*., 2009; Aranzamendi *et al*., 2011; Luyai *et al*., 2014).

Another option is to use chemoenzymatic synthesis to replicate natural glycostructural motifs and so some structures akin or identical to those of schistosomes or nematodes have been prepared, in part with our defined *C. elegans* FUT-1, FUT-6 and FUT-8 core fucosyltransferases (Yan *et al*., 2013). The resulting synthetic arrays, which can also be studied in parallel to natural arrays, have been probed with, e.g. human lectins, anti-*Schistosoma* monoclonal antibodies or with the sera of *Schistosoma*-infected humans or macaques (Brzezicka *et al*., 2015; Yang *et al*., 2017, 2018; Echeverria *et al*., 2018). Thereby, some detailed insights into recognised structures can be obtained; for instance, antibodies recognising fucosylated antennae may correlate with the stage of parasite infection, while the presence of xylose or the exact antennal N-glycan configuration may have a negative role on lectin binding or be associated with skewed IgG subtype reactivity.

## Conclusion

With this brief summary of the different categories of N-glycan modifications in invertebrates, we hope the reader will appreciate the great glycomic variety. Comparing parasitic, non-parasitic and host species is still far from complete; thus, it is still difficult to state whether certain N-glycans or epitopes are themselves hallmarks for parasitism or tropism. It may well be that each parasite has adopted aspects of its ancestors' or its hosts' glycomic capacity in order to fill a specific patho-ecological niche. On the other hand, knowledge about the glycomic status of hosts for recombinant protein production (e.g. insect cell lines) is important before, or may even aid, their use as factories for production of vaccines against parasites. In any case, only carefully performed glycomics can yield the deepest knowledge about invertebrate glycans, including exclusion of host glycans from the analyses, and is a pre-requisite for binding and other functional studies. Here, one challenge is to isolate sufficient natural glycans from parasites or related species or to recreate the structures *in vitro*. Another is to identify relevant ‘glycozyme’ genes, which will allow more recombinant glycosyltransferases to be used in chemoenzymatic synthesis and, as CRISPR/Cas9-based genetic engineering is beginning to be used in metazoan parasites (Gang *et al*., 2017; McVeigh and Maule, 2019), enable the switching on/off of certain glycosylation pathways. Indeed, a mix of analytical, biological and chemical tools will certainly prove valuable in the future to not only define the binding partners of specific glycans, but to predict their wider evolutionary occurrence and determine their function in host-parasite interactions.
